# Mutations in Influenza A Virus (H5N1) and Possible Limited Spread, Turkey, 2006

**DOI:** 10.3201/eid1403.061237

**Published:** 2008-03

**Authors:** Ender Altiok, Fulya Taylan, Osman Ş. Yenen, Gülşah Demirkeser, Mürvet Bozaci, Derya Önel, Birsen Akcadag, A. Selma Iyisan, Meral Ciblak, Emel Bozkaya, Sirin Yuksel, Selim Badur

**Affiliations:** * Acibadem Genetic Diagnosis Center, Istanbul, Turkey; † Boğaziçi University, Istanbul, Turkey; ‡ Istanbul University School of Medicine, Istanbul, Turkey; § Pendik Veterinary Control and Research Institute, Istanbul, Turkey

**Keywords:** Avian flu pandemic, H5N1, pandemic, mutation, outbreak, Turkey, dispatch

## Abstract

We report mutations in influenza A virus (H5N1) strains associated with 2 outbreaks in Turkey. Four novel amino acid changes (Q447L, N556K, and R46K in RNA polymerase and S133A in hemagglutinin) were detected in virus isolates from 2 siblings who died.

Influenza A virus (H5N1) is the predominant candidate for a future influenza pandemic if it develops efficient ability for human-to-human transmission. Since 2003, a total of 109 human deaths have been associated with this strain (*1*). Monitoring the genetic structure of this virus is needed for predicting changes that may confer ability to cause pandemics: pathogenicity, host range, and antigenic drift.

Recently, 2 avian influenza A (H5N1) outbreaks occurred in Turkey. The first outbreak, in Balikesir in northwestern Turkey in October 2005, was limited to poultry. The second outbreak, in Dogubeyazit in northeastern Turkey in November 2005, involved poultry and humans. As of January 2006, a total of 12 human cases, 4 fatal, have been confirmed (*1*).

## The Study

We analyzed molecular evolution of the virus genome by sequencing the hemagglutinin (HA), RNA polymerase (PB2), and matrix 2 (M2) genomic segments of 4 chicken and 2 human viral isolates. During the first outbreak, the MYS viral isolate was obtained from a chicken. During the second outbreak, viral isolates SU, 13, and 20 were obtained from chickens in southeastern Turkey, Anatolia, and Istanbul, respectively. Human viral isolates FK and MAK were obtained from 2 siblings who died shortly after the second outbreak. Changes in the viral genomes are summarized in the Table.

**Table:** Mutations in avian and human influenza A virus (H5N1) isolates from Turkey*

Isolate	Mutations		
	HA	PB2	M2
FK†	D158, S227	E627K, Q447L	I28V, I42T
MAK†	D158N, S227N	E627K, R46K, Q447L, N556K	I28V, I42T
MYS‡	D158, S133A, S227		I28V
SU§	D158, S227	E627K	I28V, I42T
13§	D158, S227	E627K	I28V, I42T
20§	D158, S227	E627K	I28V, I42T

*HA, hemagglutinin; PB2, RNA polymerase; M2, matrix 2 protein. †Human isolates from the second outbreak. ‡Chicken isolate from the first outbreak. §Chicken isolates from the second outbreak.

Amino acids at positions 16, 13, 18, 20, 28, 55, and 78 in M2 are associated with host specificity; those at positions 31, 34, 26, 27, and 30 are associated with resistance to adamantanes (*2*). All isolates in our study showed greatest homology to the M2 region of influenza A virus A/bar-headed goose/Qinghai /59/05 (H5N1) (GenBank accession no. AAZ16311). None of the isolates had amino acid changes associated with resistance to adamantanes. All chicken and human isolates from the second epidemic had a unique mutation (I42T) in the M2 region. Thus, the only difference between isolates from the 2 outbreaks is a mutation (T42) in isolates from the second outbreak.

The HA sequence of MYS showed 98% homology with that of influenza A virus A/chicken/Kurgan/3/2005 (H5N1) (GenBank accession no. DQ323672) and contained a unique mutation (S133A) near receptor binding residue GVSSAC at positions 134 through 139. HA cleavage sites of all isolates from Turkey contained the sequence PQGERRRKKRGLF, similar to that of influenza A virus A/duck/Novosibirsk/ 56/2005(H5N1) (accession no. ABB17275), which indicates their high virulence. MAK contained mutations D158N and S227N in the HA region. These 2 mutations are important because D158N results in a potential glycosylation site and S227N enhances affinity for human receptors.

Enhanced pathogenic potential of all avian and human influenza virus isolates from the second outbreak was shown by mutation E627K in the PB2 region. In addition, unique amino acid changes found only in human isolates were Q447L in FK and Q447L, R46K, and N556K in MAK.

## Conclusions

The Q447L and E627K mutations in FK and MAK virus isolates indicate a common origin of viruses in the 2 siblings. Unique mutations (D158N and S227N in HA and N556K and R46K in PB2) in only the MAK isolate suggest virus evolution in 1 patient. Human-to-human transmission and adaptation of the virus to infect humans have been suggested (*3*). Incomplete knowledge of the history of infections in humans and lack of sample availability limited our study. Unique mutations in PB2, particularly in these human isolates, emphasize the need for clinical, epidemiologic, and molecular studies for further understanding of the global pattern of evolution of influenza A virus (H5N1).

The close location of mutation T42 to viral regions associated with host specificity and resistance to adamantanes and isolation of virus with this mutation from humans should be studied. Although isolates from the FK and MAK group have not been available for comparison, the unique changes should be studied with respect to their ability to confer survival advantage to virus in humans.
